# Putting ‘Emotional Intelligences’ in Their Place: Introducing the Integrated Model of Affect-Related Individual Differences

**DOI:** 10.3389/fpsyg.2018.02155

**Published:** 2018-11-14

**Authors:** David J. Hughes, Thomas Rhys Evans

**Affiliations:** ^1^Alliance Manchester Business School, The University of Manchester, Manchester, United Kingdom; ^2^School of Psychological, Social and Behavioural Sciences, Coventry University, Coventry, United Kingdom

**Keywords:** emotional intelligence, individual differences, emotion, intelligence, personality, emotion regulation

## Abstract

Numerous individual differences, models, and measures have been associated with the ‘emotional intelligence’ (EI) label. This paper discusses one of the most pervasive problems regarding EI-related individual differences, namely, the lack of a meaningful theoretical framework. First, drawing upon existing theoretical frameworks, we argue that EI-related characteristics can be considered constituents of existing models of cognitive ability (ability EI), personality (trait EI), and emotion regulation (EI competencies). Second, having differentiated between these perspectives (ability, personality, and emotion regulation), we draw upon existing theory and research to build the Integrated Model of Affect-related Individual Differences (IMAID), which provides an initial mechanistic representation that explains how the different EI-related constructs are likely to interrelate and coalesce to influence affective outcomes. In essence, the IMAID is an integrated mediation model in which emotion regulation mediates the effects of ability EI and affect-related personality traits upon outcomes. Viewing EI-related constructs as interrelated extensions of well-established individual difference frameworks clarifies some pervasive misconceptions regarding EI-related characteristics and provides scholars and practitioners with a clear and useful theoretical framework ripe for exploration. We conclude by using the IMAID to suggest a theoretically driven agenda for future research.

## Introduction

**FIGURE 1:**
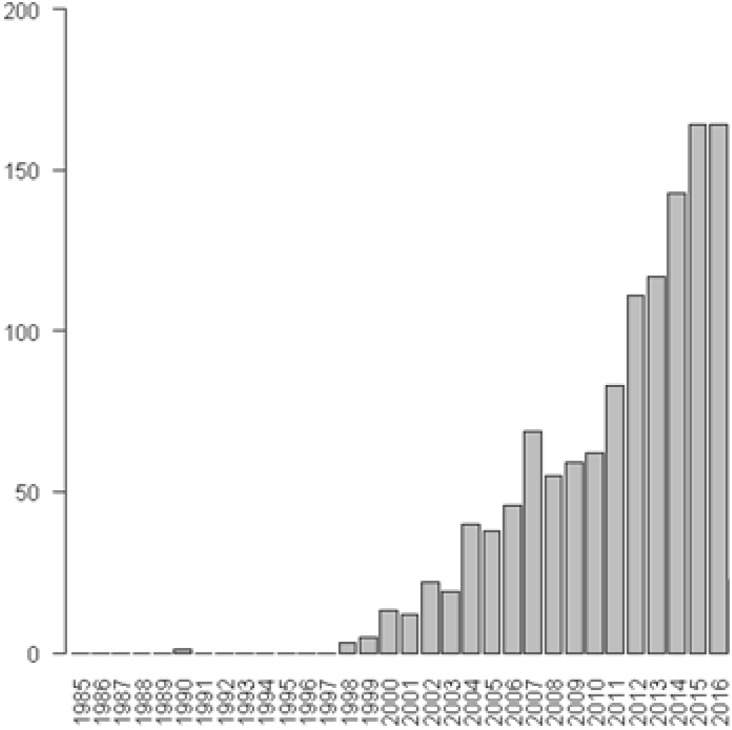
Prevalence of the term ‘emotional intelligence’ in journal articles hosted in the PubMed database between 1985 and 2016.

Emotional intelligence (EI) is a label assigned to a wide array of individual differences that has been widely adopted by scholars and practitioners. Despite this popularity, numerous concerns regarding the theoretical nature of EI-related constructs remain. Taken literally, EI should be a combination of emotion and intelligence. Emotions are considered affective episodes with a perceptual or intellectual component (e.g., perception and appraisal of emotional cues) that hold the property of intentionality (e.g., jealousy *toward* another or shame *toward* oneself; Mulligan and Scherer, 2012). Emotions are often triggered and guided by at least one appraisal of a stimulus (e.g., an impending exam) and are associated with physiological and/or cognitive change(s) (e.g., increased heart rate and self-doubting thoughts; Mulligan and Scherer, 2012). Intelligence is defined as a “mental capability that… involves the ability to reason, plan, solve problems, think abstractly, comprehend complex ideas, learn quickly and learn from experience” (Gottfredson, 1997, p. 13). Adopting this emotion plus intelligence (or cognitive ability) perspective, Salovey and Mayer (1990, p. 189) defined EI as the cognitive abilities required to “monitor one’s own and others’ feelings and emotions, to discriminate among them and to use this information to guide one’s thinking and actions”. Salovey and Mayer’s (1990) conceptualization of EI was discussed within academic circles but a more generalized version of EI was rapidly adopted following the publication of Goleman (1995) “Emotional Intelligence: Why it can matter more than IQ”. Figure 1 shows this surge in interest in terms of the prevalence of the term ‘EI’ within journal articles.

The popularized notion of EI included constructs not captured by Salovey and Mayer’s (1990) cognitive ability model, including motivation, empathy, social skills, happiness, and achievement-orientation, amongst others (Goleman, 1995; Bar-On, 1997). In response, EI researchers diversified, developing a myriad of substantively different definitions and measures – all under the EI label (Locke, 2005). The rapid and piecemeal development of EI measures outstripped meaningful theoretical advancements and the commercialization of EI tools exacerbated inconsistencies in terminology, measurement, and empirical findings (Locke, 2005; Zeidner et al., 2008).

It is not uncommon for newly proposed constructs to outstrip meaningful theoretical development (Shaffer et al., 2016), partly because producing construct labels, definitions, and measures is easier than developing meaningful theory, and partly because the latter is based upon the former. Thus, the fact that we now have many conceptualizations and operationalizations of EI is not necessarily a problem. Indeed, it is argued that “when authors begin to map out the conceptual landscape of a topic they should err in favor of including too many factors, recognizing that over time their ideas will be refined” (Whetten, 1989, p. 490). The numerous conceptualizations of EI each have their merits but growing concerns regarding their theoretical status can no longer be left unchecked (Locke, 2005; Zeidner et al., 2008). Paramount amongst these concerns are questions regarding what EI actually is, the extent to which the different EI-related constructs are distinct, and which constructs, if any, are redundant manifestations of ‘old wine in new bottles’ (Locke, 2005; Zeidner et al., 2008).

The lack of clarity surrounding EI has led some to dismiss certain elements of the EI literature, and some to dismiss EI completely (Ashkanasy and Daus, 2005; Locke, 2005). We too are skeptical of EI but believe that calls for complete rejection are premature. Before we can confidently reject or retain the various conceptualizations of EI, we need two things: clear and concise definitions that provide clear boundaries for each EI-related construct, and a theoretical framework that describes how they relate to each other and to the broader individual differences arena. Only with such an integrative theoretical framework can we determine whether EI has any scientific value.

Previous papers have provided taxonomies of EI-related constructs aimed to prevent researchers and practitioners from generalizing across the different ‘types of EI’ (e.g., Ashkanasy and Daus, 2005; Van Rooy et al., 2005; Zeidner et al., 2008). Typically, these taxonomies have been based upon measurement tools, measurement approaches (e.g., maximal performance vs. self-report), or have focused on differentiating ability EI from other models (e.g., Ashkanasy and Daus, 2005; Zeidner et al., 2008; Joseph et al., 2015). Each of these approaches has been useful, but none has yet developed into a meaningful theoretical framework that can be used to rigorously evaluate the full range of EI-related constructs or prevent conceptual confusion. Indeed, some classifications, such as the ‘mixed model’ grouping, which is atheoretical by definition, is confusing, of little scientific utility and arguably exacerbates rather than reduces conceptual confusion and false claims (e.g., Joseph et al., 2015). In contrast, we aim to proffer a framework that draws not from the inherent quirks of EI measures but from the wider individual differences literature. Specifically, in the first part of the paper we argue that EI-related constructs can be accommodated within well-established theoretical frameworks that describe cognitive ability, personality, and emotion regulation. In doing so, we remove the need for terms such as ‘mixed EI,’ and provide a simple but powerful framework to classify EI-related constructs. Having introduced this theoretically informed classification, the second contribution of the paper is to present the Integrated Model of Affect-related Individual Differences. This model provides an initial mechanistic representation that explains how the different EI-related constructs are likely to interrelate and coalesce to influence affective outcomes. By differentiating and integrating EI-related individual differences, we hope to provide scholars and practitioners with a clear and useful framework that will provide a platform for theoretical refinement, measure development, and future research.

## Perspective 1: Emotional Intelligence as Cognitive Abilities

The theoretical backdrop to ability EI research is synonymous to that of general cognitive abilities, essentially, that individuals differ in their cognitive capacity to recognize, comprehend, and manage emotions in much the same way as individuals vary in their capacity for numerical reasoning or spatial awareness (Mayer et al., 2001). However, the existence of ability EI as a form of *intelligence* has been the subject of contentious debate (Mayer et al., 2001; Locke, 2005). The two major criticisms have been philosophical (e.g., are emotions rational/logical processes?) and measurement-based (e.g., can a question on emotion have an objectively ‘correct/incorrect’ answer?). These two points of debate have been fiercely argued elsewhere and so we do not revisit them here (c.f. Bowman et al., 2002; Locke, 2005; Zeidner et al., 2008; Mestre et al., 2016). Instead, we examine the empirical evidence for considering ability EI as a constituent of broader models of cognitive ability, such as the Cattell–Horn–Carroll model (Schneider and McGrew, 2018).

Ability EI research has drawn predominantly on the Salovey and Mayer (1990) model and operationalized the construct using the Mayer–Salovey–Caruso Emotional Intelligence Test (MSCEIT; Mayer et al., 2002; formerly the MEIS; Mayer et al., 1999). This model proposes four emotion-related abilities, namely, the *perception* (accurately perceiving emotions), *facilitation* (using emotions to aid performance), *understanding* (comprehending how emotions arise and develop), and *management* (regulating one’s own or others’ emotions) of emotions (Mayer and Salovey, 1997).

The MSCEIT has been the most popular measure of ability EI and the measure and construct are often considered synonymous. Researchers have recently acknowledged that additional measures of ability EI are needed, in part because a single measure is unlikely to provide sufficient evidence for the existence of the construct (Mestre et al., 2016) and also because there have been a number of notable critiques regarding the psychometric properties of the MSCEIT (e.g., Matthews et al., 2004; Maul, 2012). For example, the perception factor has demonstrated inconsistent correlations with other measures of emotion recognition (e.g., Matthews et al., 2003; Roberts et al., 2006) and the facilitation branch does not hold in factor analyses, with facilitation measures loading on to the perception and management factors (e.g., Roberts et al., 2006; Fan et al., 2010). Indeed, numerous studies now provide support for a hierarchical three-factor model, with emotion perception, understanding, and management correlating highly and loading onto a single higher-order ability EI factor (Fan et al., 2010; MacCann et al., 2014).

Despite the measurement problems, evidence in favor of an affect-related strand of intelligence is mounting. There is clear evidence that individuals consistently differ in their ability to perceive and understand emotions (Mestre et al., 2016). Further, both the general ability EI factor and the three sub-factors are strongly correlated with measures of cognitive ability but remain somewhat distinct (Van Rooy and Viswesvaran, 2004; MacCann, 2010; MacCann et al., 2014). The most robust investigation of ability EI within the cognitive ability domain comes from MacCann et al. (2014) who tested various factor models examining the structural relations of ability EI tests and tests of general cognitive ability. The best fitting models all situated ability EI-loaded by tests of emotion perception, understanding and management – as a second-stratum factor of general intelligence alongside fluid intelligence, crystallized intelligence, quantitative reasoning, visual processing, and broad retrieval ability. Across the different factor models, ability EI typically loaded onto g at around 0.80, a similar magnitude to the other broad domains of cognitive ability tested (MacCann et al., 2014: Table 6). MacCann et al. (2014) concluded that ability EI was best considered as a sub-domain of general cognitive ability within a broad Cattell–Horn–Carroll (CHC) model of intelligence (Schneider and McGrew, 2018). Further, emerging data suggests that the various EI branches have developmental trajectories similar to closely related cognitive abilities. For example, emotion perception decreases in adulthood as do to other sensory-modality abilities, and emotion understanding/management continues to increase across the lifespan akin to other knowledge-like abilities (Mestre et al., 2016).

Although ability EI can be considered a sub-factor of existing general cognitive ability models, that does not mean it is redundant or lacking in utility. Indeed, evidence suggests that ability EI predicts a number of important outcomes when controlling for general cognitive ability and other individual differences (e.g., Van Rooy and Viswesvaran, 2004; Newman et al., 2010), with the incremental prediction most pronounced when the outcomes examined are emotion-laden (e.g., job performance in roles requiring high emotional labor; Joseph and Newman, 2010).

In sum, ability EI, or individual differences in the ability to recognize, understand, and (knowledge of how to) manage emotions appear to exist and fit within a broader cognitive ability framework (MacCann et al., 2014; Mestre et al., 2016). Ability EI shares numerous features with other elements of cognitive ability and provides useful information in explaining emotion-laden outcomes (Mayer et al., 2001; Van Rooy and Viswesvaran, 2004; Joseph and Newman, 2010). The evidence summarized here supports a conclusion that ability EI can be considered a sub-factor of general cognitive ability.

## Perspective 2: Emotional Intelligence as Personality Traits

The second perspective on EI was pioneered by Petrides and colleagues and is termed ‘trait EI.’ The definition of trait EI has evolved over time, from a construct that represents “behavioral dispositions and self-perceived abilities” (Petrides and Furnham, 2001, p. 426), to “emotion-related dispositions” (Petrides et al., 2007b, p. 273), and most recently to the “constellation of emotional self-perceptions located at the lower levels of personality hierarchies” (Petrides, 2010, p. 137). A large body of evidence demonstrates that trait EI is unrelated to ability EI (correlations are often near-zero; Van Rooy et al., 2005; Petrides et al., 2007a). Thus, we can say that trait EI and ability EI represent two distinct perspectives on EI, with ability EI linked to individual differences in intelligence and trait EI linked to individual differences in personality. However, there remains uncertainty regarding the extent to which trait EI replicates traits within existing personality models or captures a new dimension of personality.

Personality refers to the relatively stable traits that influence a person’s typical pattern of thinking, feeling, and behaving (Hughes and Batey, 2017). Given this, trait EI should refer exclusively to typical affective tendencies and not self-perceived abilities, which are distinct from personality (Chamorro-Premuzic and Furnham, 2004). However, as noted above, trait EI has shifted over time from a mixed construct (dispositions and self-perceived abilities) to a personality construct and it seems that current measures of trait EI still contain questions concerning self-perceived abilities (e.g., I would describe myself as a good negotiator; I believe I am full of personal strengths). This is not surprising given that the trait EI program did not start out to identify the “comprehensive representation of the affective aspects of personality” (Petrides et al., 2016, p. 336) that it now claims to capture. Nevertheless, future research needs to refine trait EI measures so that they focus exclusively on personality.

The most popular measure of trait EI, the TEIQue (Petrides, 2009), has a general factor that can be broken into four-sub-factors and a further 15 facets, two of which are considered auxiliary facets that do not load onto any of the four sub-factors (see Table 1). Early exploratory factor analytic evidence showing that some trait EI facets formed a factor separate to the Big Five (Petrides and Furnham, 2001; Petrides et al., 2007b), combined with evidence of modest incremental prediction (beyond short measures of the Big Five; Petrides et al., 2007a), was interpreted as indicating the discovery of a major new personality dimension (Petrides et al., 2007b; Pérez-González and Sanchez-Ruiz, 2014).

However, later research demonstrated substantial overlap between existing personality measures and trait EI measures (e.g., 57% of trait EI variance is accounted for by the Big Five factors), suggesting that trait EI was not that new or that major (Pérez-González and Sanchez-Ruiz, 2014). Indeed, when examining Petrides et al.’s (2007b, Table 4) joint factor analysis of the TEIQue and a measure of the Big Five, there is substantial overlap. Five trait EI facets had no substantial loading on the trait EI factor but did load substantially on other Big Five factors. Six trait EI facets either had their primary loading on a Big Five factor or had substantial loadings on both trait EI and one of the Big Five. Four trait EI facets loaded primarily onto the trait EI factor and had no meaningful cross-loadings. So, of the fifteen TEIQue facets, five are best considered markers of the current Big Five, and a further six can quite easily be incorporated within the Big Five model. What this analysis reveals is that the vast majority of trait EI facets are best considered markers of the Big Five, not as markers of a new trait construct. Indeed, based on a qualitative review of item content, we have illustrated further overlap between trait EI and the Big Five within Table 1.

**Table 1:** TEIQue facets and similarities to the Big Five.

TEIQue sub-factor and facets	Similar constructs found within the NEO PI-R
Emotionality	
Emotion perception	Feelings (O)
Trait empathy	Tender-mindedness (A)
Emotion expression	Hostility (N), anxiety (N)
Relationships	–
Self-control	
Emotion regulation	–
Stress management	Vulnerability (N), anxiety (N)
Impulsiveness	Impulsiveness (N)
Sociability	
Assertiveness	Assertiveness (E)
Emotion management	–
Social awareness	–
Self-esteem	Competence (C)
Well-being	
Trait happiness	Positive emotions (E), depression (N)
Trait optimism	–
Auxiliary facets	
Adaptability	–
Self-motivation	Achievement striving (C)


Despite the substantial overlap between trait EI models and the Big Five, Petrides et al.’s (2007b) analyses suggest that four facets (social awareness, emotion management, emotion expression, and trait empathy) are unique from the Big Five. In addition, our qualitative review suggests that some facets do not have direct equivalents in extant models. Thus, it is possible that trait EI research has identified meaningful personality traits that can inform and expand existing personality models. This is especially useful given that current omnibus models of personality (e.g., the Big Five) are not comprehensive in their coverage of the personality sphere (Hughes and Batey, 2017), and this is particularly true for tendencies relating to positive affect (e.g., Pytlik Zillig et al., 2002). Indeed, a number of the ‘unique’ trait EI facets, representing positive affect or low neuroticism, are not currently captured by the Big Five (e.g., emotion regulation, adaptability, and optimism). This might explain why the factors of well-being and self-control, which subsume these facets, often provide incremental prediction when examined alongside existing personality measures (Andrei et al., 2016).

So, trait EI measures capture a number of affect-related or affect-laden personality traits that span the whole spectrum of personality (i.e., facets from each of the Big Five and seemingly beyond). Having measures that provide a “comprehensive representation of the affective aspects of personality” (Petrides et al., 2016, p. 336) is undoubtedly useful for both research and practice (i.e., identifying which facets to measure during employee selection, Hughes and Batey, 2017). However, whether existing trait EI measures achieve this is debatable. The research that is now needed to achieve comprehensive coverage of affect-related personality facets involves two major steps. First, research must identify which facets of trait EI models and measures are unique and which are redundant. Second, existing personality measures (i.e., Big Five measures) need to be examined in order to identify other affect-related facets (e.g., anxiety and warmth) that are currently absent from trait EI models.

In sum, whilst trait EI research is not as clear-cut as ability EI research, we can draw several important conclusions, namely that trait EI is distinct from ability EI and that trait EI is, in essence, a collection of affect-related personality traits. In order to make this overlap and theoretical perspective explicit, from this point, we refer to this perspective without using the term ‘intelligence.’ Instead, we refer to this perspective as ‘affect-related personality.’ Using a unique label was not a lightly taken decision because whenever novel terminologies are introduced the risk of confusing matters increases. We considered the term ‘personality trait EI’ or retaining ‘trait EI.’ However, on reflection, we considered that it would be more confusing to use term ‘intelligence’ when referring to a collection of personality traits.

## Perspective 3: Emotional Intelligence as Emotion Regulation

The third broad grouping of EI-related constructs commonly discussed pertains to Emotional and Social Competencies (e.g., Goleman, 1995; Boyatzis, 2009). EI competencies are “observed when a person demonstrates… self-awareness, self-management, social awareness and social skills at appropriate times and ways in sufficient frequency to be effective in the situation” (Boyatzis et al., 2000, p. 344). This definition is so broad that almost any intrapersonal or interpersonal behavior could be classified under this definition, and many have been. Indeed, EI competency models include constructs synonymous with personality (e.g., conscientiousness, optimism; Joseph et al., 2015), constructs concerning the regulation of one’s own and others’ emotions (e.g., emotional self-control, empathy, and conflict management) and a broad array of performance outcomes (e.g., teamwork, service orientation, innovativeness, social responsibility, leadership; Bar-On, 1997; Boyatzis et al., 2000; Boyatzis, 2009).

The varied nature of these models has led numerous authors to refer to them as ‘mixed models’ (e.g., Joseph et al., 2015), with mixed models frequently and justifiably criticized for their lack of theoretical clarity (Mayer et al., 2000; Daus and Ashkanasy, 2003; Ashkanasy and Daus, 2005; Locke, 2005; Zeidner et al., 2008). Indeed, when a construct is so broad that it can reasonably accommodate almost everything it is essentially protean and thus meaningless (Hughes, 2018). ‘Valid’ constructs have clear definitions, clearly defined content, and clear boundaries (Hughes, 2018). Competency EI models have none of these features (Locke, 2005; Zeidner et al., 2008). In addition, competency measures share larger correlations with measures of other constructs than each other (Brackett and Mayer, 2003), have low internal consistency and test–retest reliability (Zeidner et al., 2008), incoherent and inconsistent factor structures (Livingstone and Day, 2005), and when considered alongside personality, intelligence, and self-perceptions, have little predictive value (Joseph et al., 2015). Given the lack of supporting evidence (and quite damning critical evidence), we concur with the previously espoused views that EI competencies represent a proportion of the EI literature that could be abandoned (e.g., Mayer et al., 2000; Brackett and Mayer, 2003; Daus and Ashkanasy, 2003; Zeidner et al., 2004, 2008; Ashkanasy and Daus, 2005; Locke, 2005). Indeed, the continued use of EI competency models is likely to do “much more harm than good” (Daus and Ashkanasy, 2003, p. 70) when seeking to build coherent theoretical accounts of affective processes and related individual differences.

Although we see no meaningful future for current EI competency models, we do see that the general aim of assessing emotional competence and integrating it within a broad framework of emotion-related individual differences is of value. As noted above, the constructs included within competency models span three domains: personality, broad performance outcomes, and emotion regulation. Within the delineation of emotion-related constructs we present, the personality component is captured under the ‘affect-related personality’ (e.g., trait EI) banner, so any personality constructs here would represent construct proliferation, and the broad performance outcomes cannot reasonably be considered affect-related individual differences. However, we would argue that the elements related to emotion regulation do have a place within the broad domain of affect-related individual differences. Thus, we would prefer to see such work move away from EI competencies and focus instead on emotion regulation, which represents ‘the use of strategic cognitions or behaviors to improve or worsen [one’s] own feelings and those of other people, in the pursuit of hedonic, relational and instrumental goals’ (Niven et al., 2011, p. 71). In essence, emotional competence and emotion regulation are aiming to address the same phenomenon, namely, using emotions to facilitate goal attainment. In Table 2, we have noted some clear areas of overlap between emotion regulation models and EI competencies.

**Table 2:** Overlaps between emotion regulation classes, emotion regulation strategies, and outcomes currently considered to be emotional intelligence competencies.

Emotion regulation class (Gross, 2015)	Example emotion regulation strategies (Peña-Sarrionandia et al., 2015)	Example emotional intelligence competency (Boyatzis, 2009)
Situation selection	Avoidant-coping Forecast accuracy	Emotional self-awareness Empathy
Situation modification	Conflict resolution Social support search	Conflict management Influence
Attentional deployment	Rumination Distraction	
Cognitive change	Positive reappraisal Humor	Optimism/positive outlook
Response modulation	Venting/suppression Substance use	Emotional self-control


Given that EI competencies and emotion regulation are both concerned with the same phenomena and the clear overlap in core constructs (see Table 2), it makes sense to consolidate the two fields to avoid construct proliferation (i.e., the proposition and marketing of multiple ostensibly unique but actually largely overlapping constructs). This consolidation is likely, in our view, to lead to the abandonment of EI competencies in favor of emotion regulation for three main reasons:

First, emotion regulation has well-developed theories such as the process model of emotion regulation (Gross, 1998, 2015). According to this model, there are five classes of emotion regulation: (i) choosing situations to engage with or avoid (situation selection), (ii) modifying that situation (situation modification), (iii) directing attention within the situation (attentional deployment), (iv) attributing a meaning to the situation (cognitive change), or (v) altering the response to the situation (response modulation). At all five stages, different emotion regulation strategies can be adopted and if implemented successfully can facilitate goal-attainment (Gross, 2015). Some example strategies are displayed in Table 2. The process model of emotion regulation has recently been extended to consider a significant gap noted by the model’s author, namely, how individuals come to choose which emotion regulation strategy to adopt. Specifically, Gross proposed three key antecedents to the enacting of emotion regulation, namely, identification, selection, and implementation (Gross, 2015). As we will discuss later, we see a prominent role for ability EI and affect-related personality traits in explaining individual differences in identification (i.e., how capable/prepared people are to pay attention to emotions), selection (i.e., the ability to identify an appropriate emotion regulation class), and implementation (i.e., the specific behavioral nuances with which people enact their regulation).

At this point, it is worth noting how emotion regulation differs from ability EI and in particular the management branch, which is sometimes referred to as the regulation branch (Joseph and Newman, 2010, 2015). Emotion regulation as we have noted above refers to processes and behaviors regarding the up-regulation and down-regulation of emotions. The emotion management branch of ability EI refers to crystallized knowledge regarding these processes and behaviors. In other words, the difference between knowing that consuming alcohol will not improve one’s mood, and drinking it regardless. The two are interrelated, but they are not synonymous, one is knowledge and the other is action.

Second, although EI competency models refer to broad outcomes (e.g., optimism or influence), emotion regulation models focus on the specific strategies utilized (see Table 2). For example, optimism can be obtained and maintained through positive reappraisal and the use of self-enhancing humor (Scheier and Carver, 1987), whilst influence can be gained in a number of ways, including appropriate displays of anger (e.g., Sy et al., 2005; Côté and Hideg, 2011). This focus on specific strategies provides a greater insight into the processes behind affective phenomena (Mestre et al., 2016) and thus can lead to more specific theories and useful practical guidance (i.e., training programs).

Third, emotion regulation measures are superior to EI competency measures in terms of theoretical coherence and psychometric properties (c.f., Matthews et al., 2003; Bridges et al., 2004). For example, one of the most widely used measures, the Emotion Regulation Questionnaire (ERQ; Gross and John, 2003) differentiates between re-appraisal and suppression strategies guided by theoretical models (Gross, 1998; Gross and John, 2003). In addition, the ERQ scales demonstrate adequate reliability, a stable factor structure, and provide prediction of numerous socially important outcomes (e.g., depression, anxiety, stress, interpersonal functioning, well-being, social adjustment, and decision-making; see Spaapen et al., 2014).

In sum, both emotional competence and emotion regulation are attempting to address the same phenomenon: using emotions to facilitate goal attainment. However, only emotion regulation actually achieves this. Compared with EI competency models, emotion regulation models are built on stronger theory, contain more specific construct identification, inspire better measures, and show better predictive properties. Thus, we believe that researchers interested in emotional competence or skill should avoid EI competency/mixed models and instead focus on models of emotion regulation. The integration of EI research with emotion regulation echoes a call from Mestre et al. (2016, p. 327) who recently stated that, ‘studying EI through the theoretical framework of emotion regulation may produce greater understanding of the mechanisms by which EI capacities influence valued outcomes.’ We agree with Mestre et al. (2016) and see great value in the integration of EI and emotion regulation (see also Hughes and Evans, 2016). In the next section, we expand upon these claims by building an integrative model that combines ability EI, affect-related personality traits, and emotion regulation.

## An Integrated Model of Affect-Related Individual Differences

Using the aforementionedtheoretically informed classification, the second major goal of this paper is to introduce the Integrated Model of Affect-related Individual Differences. This model aims to provide an initial mechanistic representation that explains how the different EI-related constructs are likely to interrelate and coalesce to influence affective outcomes. By differentiating and integrating EI-related individual differences, the current paper aims to provide scholars and practitioners with a clear and useful framework that will provide a platform for theoretical refinement, measure development, and future research.

Previous influential models or classifications of EI-related constructs (e.g., trait vs. ability; stream 1, 2, and 3; ability vs. mixed) have typically been based on sub-optimal definitions or measurement tools (e.g., Ashkanasy and Daus, 2005; Zeidner et al., 2008; Joseph et al., 2015). For example, Ashkanasy and Daus (2005) influential classification proposed three streams each based on a certain measurement approach, namely, ability EI tests, self- or other-reported ability EI, and EI competency measures. Whilst such groupings have utility in distinguishing among measures, they are, by nature, atheoretical and fail to provide a solid platform for theoretical development. Equally, previous reviews have tended to adopt an ability EI vs. others (mixed models, trait EI, competency) approach and as a result, researchers have tended to treat the different EI-related constructs as competitors. Indeed, there have been numerous papers pitting the different EI models against each other with a view to identifying which EI is the ‘correct’ or ‘best’ EI (e.g., Mayer et al., 2000; Zeidner et al., 2008).

In contrast, we have used the broader individual differences literature as the basis for our classification of EI-related constructs and were not bound by existing measurement tools or deficient definitions (e.g., mixed models). As a result, our classification addresses calls to provide clear boundaries, aligned with traditional individual differences theory, for each of the major EI-related constructs (Zeidner et al., 2004). Importantly, this means that these different EI-related constructs are no longer in direct competition but can instead be viewed as complementary. Rather than conducting predictive validity competitions, pitting different EI measures against each other and other individual differences, we can instead focus on building and testing meaningful theoretical models that explain how individual differences in ability EI, affect-related personality, and emotion regulation interrelate to influence affective behavior (e.g., Seal and Andrews-Brown, 2010; Hughes and Evans, 2016; Mestre et al., 2016). Accordingly, we now propose a model that integrates the three perspectives.

**FIGURE 2:**
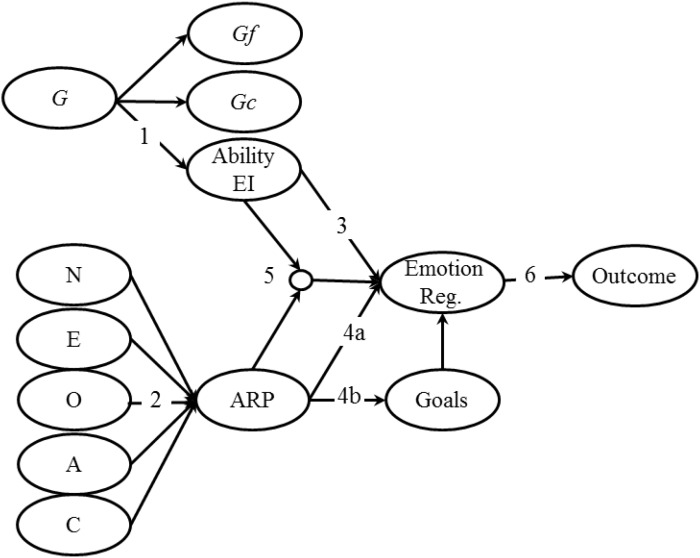
Integrated model of affect-related individual differences. G, general factor of intelligence; Gf, fluid intelligence; Gc, crystallized intelligence; N, neuroticism; E, extraversion; O, openness; A, agreeableness; C, conscientiousness; ARP, affect-related personality traits; Emotion reg., emotion regulation.

The key principles of the Integrated Model of Affect-related Individual Differences are as follows. We propose that ability EI (a sub-factor of cognitive ability) and affect-related personality traits (a collection of affect-related personality traits) drive the identification, selection, and successful implementation of various emotion regulation strategies. In turn, emotion regulation influences important outcomes. A visual representation of the Integrated Model of Affect-related Individual Differences is presented in Figure 2. In essence, this is an integrated mediation model in which emotion regulation mediates the effects of ability EI and affect-related personality traits upon outcomes (Joseph and Newman, 2010; Côté et al., 2011; Hughes and Evans, 2016; Mestre et al., 2016; Szczygieł and Mikolajczak, 2017). The model is inspired by and builds upon previous integrations of personality, intelligence, and skills that have proven successful in explaining behavior in other domains (e.g., McClelland, 1973; Zeidner, 1995; Chamorro-Premuzic and Furnham, 2004). Perhaps the key strength of the model is that it provides a testable framework that has the potential to explain how individual differences in ability EI and affect-related personality manifest in diverse behavior and differential outcomes. The model goes beyond simple descriptive correlations and direct effects that have dominated previous research and instead seeks to explain how affective outcomes arise through the dynamic interactions between affect-related individual differences. As a result, the Integrated Model of Affect-related Individual Differences focuses not simply on *what* is predicted by EI-related constructs but *how* these constructs influence outcomes. A greater understanding of how EI-related constructs interact and the nature of the mechanisms by which they influence outcomes stands to inform future theory, measurement, and intervention design.

A few previous papers have proposed and/or partially tested models that integrate some EI-related constructs (e.g., Joseph and Newman, 2010; Seal and Andrews-Brown, 2010; Peña-Sarrionandia et al., 2015; Mestre et al., 2016). However, none of these models have included all unique elements of the EI literature, all have been bound by existing measurement tools and poorly defined constructs (e.g., mixed models), and most have not considered EI-related constructs within the broader individual differences literature. For example, Seal and Andrews-Brown (2010) posit mediated and moderated relationships between three measures, which they label emotional ability (Salovey and Mayer, 1990), emotional quotient (Bar-On, 1997) and emotional competence (Goleman, 1995). The latter two are considered ‘mixed models,’ overlap substantially, and as discussed above have been heavily criticized from theoretical and empirical standpoints. This model also fails to provide theoretically meaningful links to the broader literature or draw clear boundaries separating these constructs. As a result, its utility for theory building is limited. In contrast, Mestre et al. (2016) present compelling evidence supporting the positioning of ability EI as a sub-factor of general cognitive ability. They go on to argue that emotion regulation could be the process by which ability EI influences outcomes. This link was supported by a previous meta-analysis demonstrating associations between ability EI scores and emotion regulation (Peña-Sarrionandia et al., 2015). The Integrated Model of Affect-related Individual Differences builds upon this paper positing emotion regulation as the mechanism through which EI-related constructs influence outcomes. However, it extends this discussion beyond ability EI to include affect related personality traits. Further, as we discuss in more detail below, the model also posits that individual differences in emotion regulation are most likely driven by both ability EI and affect-related personality traits (Hughes and Evans, 2016).

Overall, the Integrated Model of Affect-related Individual Differences makes several key contributions to the literature. First, by tying each EI-related construct to existing models of individual differences it provides a clear, theoretically coherent, and parsimonious description of the three key perspectives. Second, the model posits EI-related constructs not as competitors but as complementary constructs that are meaningfully entwined and which coalesce to produce emotion-relevant behavior. Third, through this integration, the model provides a framework that can explain how individual differences in ability EI and affect related personality traits influence patterns of emotion regulation and subsequently socially important outcomes. In the following sections, we discuss integration further and review extant empirical evidence in support of the key pathways hypothesized. The Integrated Model of Affect-related Individual Differences is inherently causal but most EI research is cross-sectional and correlational in design. Because such designs do not model data in a manner that is appropriate for determining causal relationships (Antonakis et al., 2010; Hughes et al., 2018), where available, studies adopting experimental designs, which are better suited to examining causality, are noted (Antonakis et al., 2010).

## Pathway 1: *G* → Ability EI

As discussed above, ability EI satisfies a number of important criteria to be considered a cognitive ability (Van Rooy and Viswesvaran, 2004; MacCann et al., 2014; Mestre et al., 2016). More specifically, current evidence suggests that ability EI is best conceived of as a second-order factor of cognitive ability that is hierarchically structured, consisting of a general factor and three sub-factors, namely, the ability to perceive, understand, and manage emotions (Fan et al., 2010; MacCann et al., 2014). Essentially, ability EI reflects the cognitive capacity to process emotion-laden information (Mayer et al., 2016).

## Pathway 2: Big Five → Affect-Related Personality Traits

Trait EI represents a compound construct containing affect-related personality traits (Petrides et al., 2007b; Petrides, 2010). Given the pervasiveness of the Big Five and Five Factor Model (FFM) within personality research, and the significant overlap between trait EI and FFM facets (see Table 1), it is appropriate to seat the affect-related personality traits perspective within this model (van der Zee and Wabeke, 2004). Historically, most affect-related personality research has focussed on global factors (e.g., total trait EI scores). However, we suggest that this is sub-optimal and potentially misleading, for two main reasons. First, compared to facets, broad factors lead to underestimates and/or distorted estimates of construct relationships (i.e., reduced predictive validity, Hughes and Batey, 2017). This is especially true when facet content is diverse (Hughes and Batey, 2017), like it is with affect-related personality traits, which span all of the Big Five. Second, factors created from a selective subset of traits (e.g., TEIQue) might well be misleading due to data pre-structuring. Briefly, factor solutions are only as strong as the variables that are entered for factoring, and factors identified can only be considered to ‘exist’ or be ‘accurate’ if they are derived from the entire domain of possible variables (i.e., all affect-related personality facets/items). Given that this was not the case for measures such as the TEIQue (which was developed based on a competency EI model), it is perhaps questionable what the global trait EI and 4 sub-factors really represent. Indeed, as we discussed, a number of TEIQue facets do not load on the general factor. Thus, we think that targeted, theory driven, facet-level analyses are the way forward (Hughes and Batey, 2017). That is, researchers should measure the affect-related personality facets that are relevant to their study. Although for slightly different reasons, this recommendation echoes recent calls from the pioneers of the personality based approach to EI (Petrides et al., 2016).

## Pathway 3: Ability EI → Emotion Regulation

Emotion regulation refers to the strategies used to adapt emotions (e.g., suppress or exacerbate) experienced by the self and others in order to facilitate goal-attainment (Niven et al., 2009, 2011; Gross, 2015). If you want to achieve the goal of increasing your positive emotions, you might tell yourself a funny joke. If you want to reduce someone else’s anxiety before a test, you might reassure them. Emotion regulation consists of three main decisions (Gross, 2015). First, a person must select if/which emotions need to be regulated in any given situation (identification). Next, the person must choose when regulation strategies (i.e., which of the emotion regulation classes, see Table 1), should be utilized (selection). Finally, in the implementation phase, the person must enact the regulation by translating the broad emotion regulation strategy (e.g., cognitive change or situation modification) into specific cognitive or behavioral strategies (e.g., positive reappraisal or conflict resolution). The identification, selection, and implementation stages were recently espoused and understanding them is likely to help explain the existence of consistent individual differences in the frequency and style of emotion regulation (Gross, 2015).

The recency with which the identification, selection, and implementation phases were espoused means that no specific theory, model, or empirical evidence describes relationships between the two. However, there is mounting evidence and increasing theoretical rationale to support a reliable link between ability EI and the use of emotion regulation strategies (e.g., Joseph and Newman, 2010; Peña-Sarrionandia et al., 2015; Hughes and Evans, 2016; Mestre et al., 2016). For example, a recent meta-analysis found a number of moderate-strong relationships between ability EI and emotion regulation strategies spanning the five major emotion regulation classes (Peña-Sarrionandia et al., 2015). As an indication of the degree of the relationship, ability EI reliably predicted the use of nineteen of the 22 emotion regulation strategies investigated (i.e., 95% confidence intervals not crossing 0). Importantly, the strength and direction of the relationships was not uniform, that is, those high in ability EI do not simply regulate more and/or use a greater number of strategies. The pattern of relationships suggests that individuals high in ability EI regulate emotions earlier (which is typically adaptive), adopt more of the strategies typically seen as adaptive (e.g., social support seeking), and fewer of those typically seen as maladaptive (e.g., rumination; Peña-Sarrionandia et al., 2015). These theoretical and empirical arguments suggest that ability EI (the cognitive capacity to process emotion-laden information) is likely to be one of the key determinants of the characteristic patterns of emotion regulation displayed by individuals (Mestre et al., 2016).

Experimental research in the field is mostly convergent, with ability EI linked to effective mood maintenance and repair following mood induction via film clips (positive and negative, respectively; Ciarrochi et al., 2000). Similarly, ability EI has been associated with lower worry and avoidant coping during stressful tasks (Matthews et al., 2006). Furthermore, using eye-tracking equipment during an experimental protocol, Davis (2018) found a general orienting preference whereby emotion management was associated with avoidance of negative emotion (anger). However, higher levels of ability EI are not always considered positive, with some relationships with higher cortisol reactivity and thus slower recovery from stress (Bechtoldt and Schneider, 2016).

**FIGURE 3:**
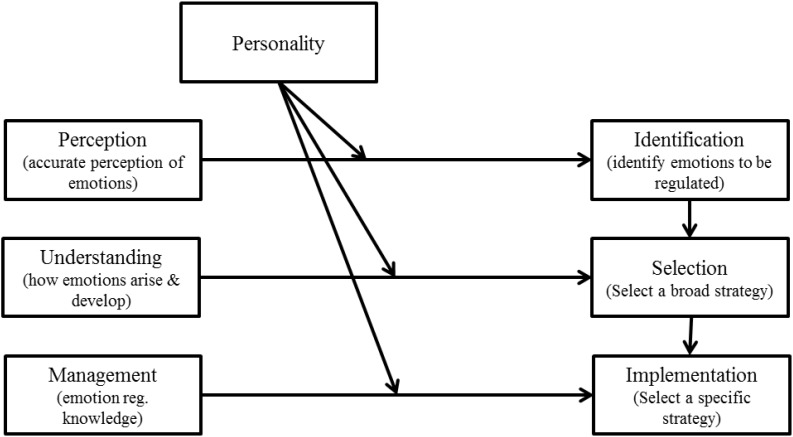
Possible interactions between the sub-factors of ability EI and personality traits in explaining the identification, selection, and implementation of emotion regulation strategies.

In sum, whilst experimental studies are rare, those available are broadly consistent with survey studies and support the notion that ability EI is likely to be one antecedent of emotion regulation style. Indeed, it would be surprising if an individual’s ability and knowledge pertaining to the perception, understanding, and management of emotions were unrelated to emotion regulation. Although speculative here, it might be expected that meaningful relationships will emerge between the perception factor of ability EI and the identification stage of emotion regulation, between understanding and selection, and between management and implementation (see Figure 3, discussed below).

## Pathway 4A and 4B: Affect-Related Personality → Emotion Regulation

Whilst ability EI correlates with emotion regulation, it alone does not explain emotion regulation (Mayer et al., 2016). There is a well-documented gap between ability and behavior; individuals with similar levels of ability EI adopt diverse emotion regulation strategies, some of which can be unproductive (e.g., Côté et al., 2011). Both theoretical rationale and empirical evidence suggest that personality also plays a role in guiding individual’s emotion regulation (Côté et al., 2011; Davis and Humphrey, 2014; Fiori, 2015; Peña-Sarrionandia et al., 2015; Hughes and Evans, 2016). Personality traits shape individuals’ preferences, attentional focus, and interpersonal behavior, all of which are likely to influence one’s choice of emotion regulation strategy (e.g., Côté et al., 2011; Peña-Sarrionandia et al., 2015). Indeed, meta-analytic estimates suggest that affect-related personality traits are associated with emotion regulation in a similar fashion to ability EI. That is, there is a consistent relationship (i.e., 35 of 37 effect sizes calculated presenting 95% confidence intervals not including 0; Peña-Sarrionandia et al., 2015). The nature of these associations suggest that those who scorer higher on measures of trait EI tend to regulate earlier and use regulatory strategies typically considered adaptive more often than strategies typically considered maladaptive. In addition, effect sizes are, on average, larger than those reported for ability EI (Peña-Sarrionandia et al., 2015). Perhaps this is not hugely surprising, in fact, it would be more surprising if personality did not shape emotion regulation style, for instance, if those high in trait optimism did not frequently use positive reappraisals and those high in trait anxiety did not frequently ruminate.

Experimental results also support a causal relationship between affect-related personality and emotion regulation. For example, trait EI was negatively related to psychological and physiological reactivity when exposed to a stressor (Mikolajczak et al., 2007) and positively related to exhibition of self-efficacy and appraising stressful situations as challenges rather than threats (Mikolajczak and Luminet, 2008). Similarly, affect-related personality traits have been associated with greater susceptibility to mood induction, moderating the effect of experimental stressors on subsequent mood deterioration (Petrides and Furnham, 2003; Mikolajczak et al., 2009).

Above, we hypothesized that there might be meaningful relationships between the three primary EI abilities (perception, understanding, and management) and the identification, selection, and implementation phases of emotion regulation. It is also likely that certain affect-related personality traits will predispose individuals to think and act in specific ways within these phases. For example, certain traits (e.g., emotion perception and empathy) might predispose individuals to invest more effort in monitoring one’s own and others’ emotions (the *identification* phase). Other traits (e.g., anxiety or optimism or emotional expression) might predispose individuals to *select* certain classes of emotion regulation (e.g., situation selection or cognitive change or response modulation) and to *implement* them using specific strategies (e.g., avoidant coping or positive reappraisal or venting). It is also likely that intrapersonal affect-related personality traits (e.g., stress management) will predict the regulation of one’s own emotions, whilst the interpersonal traits (e.g., social awareness) will predict the regulation of others’ emotions.

One of the major mechanisms through which affect-related personality traits are likely to influence patterns of emotion regulation, is goals and motives (Figure 2, path 4b). Emotion regulation is often, if not always, goal-driven (Gross, 2015) and personality shapes values, goals, and motives (Grant and Mayer, 2009; Parks-Leduc et al., 2015), with affect-related personality traits likely to influence motives and goals within affect-related settings (John and Gross, 2004; Hughes and Evans, 2016). It has been argued that the general goal of emotion-regulation should be to ‘feel good’ and thus personality is perhaps not that key (e.g., Larsen, 2000; Tice et al., 2004). However, empirical evidence does not support this assertion. Individuals demonstrate considerable variation in how they want to feel and in the direction in which they regulate their emotions (e.g., Gross et al., 2006; Tamir, 2016). For example, Heimpel et al. (2002) found that participants who were low in self-esteem and had recently experienced a failure/loss did not want to feel immediately better. In contrast, those high in self-esteem did seek to make themselves feel better. This multi-study paper showed that the varying regulatory goals were not due to differences in knowledge or expected affect changes but reflected dispositional and stylistic preferences that are almost certainly shaped, to some degree, by personality (e.g., Grant and Mayer, 2009; Augustine et al., 2010; Hughes and Evans, 2016).

We believe that a systematic empirical effort to examine the relationships between affect-related personality and emotion-regulation goals/motives would be hugely useful for theoretical development and explaining consistent individual differences within emotion regulation. A recently published taxonomy (Tamir, 2016), provides a particularly useful framework for guiding examinations between personality, goals, and emotion regulation. The framework consists of two higher-order classes of motives namely, hedonic and instrumental, which subsume six lower-order classes (see Tamir, 2016, Figure 1). These lower-order classes lend themselves to a number of hypotheses regarding affect-related personality traits. For example, trait sociability (contained with the TEIQue and most other personality models) is likely to generate goals and motives that are aligned with the social-instrumental class identified by Tamir (2016). Accordingly, one might hypothesize that those high in trait sociability will frequently be motived to regulate emotions in a social-instrumental manner through the use of emotion regulation strategies such as social support seeking and humor use. Similarly, links between trait optimism and prohedonic motives (i.e., the motive to feel good) or trait achievement striving and performance-instrumental motives could easily be theorized.

In addition, most previous examinations of EI-related constructs and emotion regulation have focussed almost exclusively upon on self-regulation (e.g., Peña-Sarrionandia et al., 2015; Mestre et al., 2016; Tamir, 2016). We believe this to be an oversight and expect that affect related personality traits will be of (equal, perhaps even greater) importance to the approaches adopted when attempting to regulate others’ emotions (Niven et al., 2011). For example, many affect-related personality traits (e.g., empathy, social awareness) drive an outward focus that is likely to produce motivations aimed at appeasing or pleasing others. Such goals are likely to affect the choices of emotion regulation strategies. For example, although venting (i.e., verbally complaining about a negative event) can be a productive self-regulation strategy, it can be counterproductive for those who receive the venting. It is possible that those high in empathy or social awareness may be more likely to consider the consequences of venting for others and thus suppress their desire to vent in order to regulate the emotions of others (Hughes and Evans, 2016). Currently, however, there is a dearth of research exploring why people engage in other-focused emotion regulation and the different styles with which they do so (Niven, 2016). Another recent taxonomy might be of use in guiding future research is Niven’s (2016) work-specific motives framework of interpersonal emotion regulation. The framework identifies eight specific motives which are subsumed under the three major needs proposed in self-determination theory. The framework shares a number of commonalities with that proposed by Tamir (2016). For example, there are different manifestations of motives depending on whether the broad goal is hedonic/pleasure or instrumental/performance focussed, these motives are hierarchically structured, and the lowest-level of abstraction provides a classification system that would lend itself to building logical hypotheses positing personality traits as antecedents of these motives.

## Pathway 5: Interaction Between Personality and Ability → Emotion Regulation

In addition to independent effects, it is also possible that ability EI and affect-related personality interact in explaining emotion regulation (Côté et al., 2011; Fiori, 2015; Hughes and Evans, 2016). For example, Côté et al. (2011) found that when individuals were high in both ability EI and moral identity, they were more likely to behave in a prosocial manner, but if they were high in ability EI and Machiavellianism, they were more likely to display interpersonal deviance. Importantly here, higher ability EI was associated with greater performance but the direction of the performance (prosociality or deviance) was explained by personality traits. Similarly, Fiori (2015) found that ability EI interacted with the personality trait of emotionality (similar to neuroticism, marked by fearfulness, anxiety, sentimentality and a dependence on social support) to predict interpersonal effectiveness as assessed through a presentation task. Here, those high in ability EI and low in emotionality (i.e., emotionally stable) were the best performers, whilst those high in ability EI and emotionality were average performers.

The above evidence demonstrates that both ability EI and affect-related personality traits are needed to explain performance. However, all of these studies omit the important mediating mechanism of emotion regulation and thus likely underestimate the effects of EI-related constructs and reduce their ability to explain how the EI-related constructs influenced the outcomes. As noted above, we also expect that ability EI and affect-related personality traits will interact to drive both the selection and implementation of differing emotion regulation strategies. For example, two individuals equally high in ability EI with differing levels of trait optimism (a facet of trait EI; Petrides et al., 2007b) might differ in the speed and frequency with which they engage in positive interpretations (see Hughes and Evans, 2016 for further discussion). Some preliminary evidence for this comes from Davis and Humphrey (2012) who found that ability EI moderated the effects of stressors upon coping strategies and that trait EI moderated the effects of coping strategies upon depression. Although not a direct test of the interactions proposed here, the results suggested that EI-related constructs do interact with some emotion regulation strategies and showed that being high in both ability EI and trait EI facilitated effective coping whereas being high in one or the other was insufficient. Essentially, there are well-established ability and stylistic elements to individuals’ patterns of emotion regulation and it is also possible that these two components can be complementary or at odds at any given stage of the emotion regulation process (Izard et al., 2008). If we consider these potential interactions further, what we might expect to see is a pattern whereby ability EI accounts for differences in knowledge/ability related to emotion regulation, personality accounts for differences in style, and the interaction between these two elements provides a meaningful insight into individual differences in emotion regulation. A graphical representation of the main proposed interaction is contained in Figure 3.

Thus, the Integrated Model of Affect-related Individual Differences presented here provides a framework to begin address numerous important questions facing emotion regulation:

“What leads a person to use one rather than another of the various emotion regulation strategies described by the process model? … the model is silent as to how these various emotion regulation strategies are actually started or stopped. What initiates emotion regulation? What directs specific emotion regulation strategies? And why do some people regulate emotions successfully while others fail to regulate emotions as they should?” (Gross, 2015, p. 9).

One interesting additional question concerns the extent to which the identification, selection, and implementation of emotion regulation strategies occurs via conscious or implicit (automatic) processes (Fiori, 2009). The Integrated Model of Affect-related Individual Differences provides a framework that could guide initial examinations into the relationships between different elements of EI and emotion regulation and the nature of the cognitive processes underlying them (Fiori, 2009).

## Pathway 6: Emotion Regulation → Outcomes

Finally, we argue that emotion regulation influences meaningful intrapersonal (i.e., calming oneself before an exam) and interpersonal (i.e., conflict resolution within a team) outcomes. There is a wealth of empirical evidence to support this claim (c.f., Gross, 2015), with emotion regulation playing “a core role in everyday social life” (Niven et al., 2012, p. 247) with utility demonstrated across social, health, educational, and occupational outcomes (Gross, 2002; Peña-Sarrionandia et al., 2015). Indeed, robust links to outcomes have been established using emotion regulation, EI competency and regulation-competency hybrid scales (e.g., Austin et al., 2010). Thus, this pathway is well supported by previous evidence. The novelty here is that we are arguing that emotion regulation does not simply occur (Gross, 2015), there are drivers of emotion regulation and those drivers are affect-related abilities and personality traits. From this perspective, we can say that emotion regulation represents the principal mediating mechanism through which ability EI and affect-related personality traits influence outcomes (Joseph and Newman, 2010; Hughes and Evans, 2016; Mestre et al., 2016).

## Implications and Future Research

The first key contribution of this article was to present a simple theoretical framework that clearly states what each EI-related construct is and is not. Specifically, that ability EI represents an extension to existing cognitive ability models, affect-related personality (e.g., trait EI) represents a collection of affective personality traits, and interest in emotional competence should focus on emotion regulation. To some researching at the cutting-edge of EI, this might seem a relatively modest extension of the extant literature. However, our reading of the EI literature suggests that it is a much-needed clarification, that explicitly demonstrates that the myriad of EI-related constructs can be accommodated within well-established individual difference frameworks, and in doing so, provides a clear theoretical base and boundaries for each. Our framework negates the need for atheoretical terms such as ‘mixed EI,’ is falsifiable, is more descriptive and explanatory in nature than previously published classifications (e.g., streams 1, 2, and 3), and is less adversarial because it does not set the different perspectives as competitors. The second notable contribution from this paper, the Integrated Model of Affect-related Individual Differences, comes from adopting these three perspectives and in drawing the different EI-related constructs together in a complementary manner. This model provides an initial mechanistic representation that explains how the different EI-related constructs are likely to interrelate and coalesce to influence important emotion-relevant outcomes through the identification, selection, and implementation of emotion regulation strategies. Accordingly, the model also provides a solid platform for empirical exploration, theoretical refinement, measure development, and possibly practical application.

Despite its promise, the Integrated Model of Affect-related Individual Differences is clearly exploratory, and needs systematic, rigorous, and detailed empirical scrutiny. For example, some of the pathways proposed (e.g., interaction between ability and personality vs. separate pathways) could be argued to represent competing explanations for the same empirical effect. Hopefully, future research will examine these competing pathways and provide evidence regarding if/when each pathway is most pertinent. Nevertheless, the evidence discussed strongly supports the major premise of the model, namely, that both ability EI and affect-related personality traits influence the selection and implementation of emotion regulation strategies (Côté et al., 2011; Davis and Humphrey, 2014; Fiori, 2015; Peña-Sarrionandia et al., 2015; Hughes and Evans, 2016). Accordingly, the review presented and the model proposed lead to a number of implications for future research.

First, calls to completely abandon all EI research (e.g., Locke, 2005) seem misguided. However, concerns regarding construct proliferation and redundancy do hold merit. Equally, no aspect of EI is the magical silver bullet often claimed (Goleman, 1995), instead, elements of the EI literature can be seen as extensions of existing ability, personality, and emotion regulation frameworks.

Second, our review of research surrounding the Integrated Model of Affect-related Individual Differences suggests that piecemeal assessment of EI related constructs will give misleading findings. Future research interested in explaining emotion regulation or broader outcomes needs to incorporate measures of ability *and* personality, and consider the interactions between these variables. Equally, studies which estimate direct effects between outcomes and ability EI or affect-related personality traits are likely to underestimate the relations due to the omission of emotion regulation as a mediator.

Third, the research reviewed and the hypotheses made in this paper suggest that focussing upon global ability EI, broad factors containing multiple affect-related personality traits, and/or broad emotion regulation measures is likely to hide important nuanced relationships. Accordingly, we suggest that future researchers adopt a more specific approach measuring theoretically relevant abilities, personality traits (or facets), and regulation strategies. This call echoes similar calls in other applied domains (e.g., individual differences at work, Hughes and Batey, 2017) and calls made from prominent EI and emotion regulation researchers (e.g., Gross, 2015; Mestre et al., 2016; Petrides et al., 2016).

Fourth, although our focus in this paper is not on measurement tools, our review has several implications for the nature of the constructs referred to under the EI banner and how they are operationalized. With regard to ability EI, we noted a number of psychometric misgivings with existing measures (i.e., lack of reliability, inconsistent factor structure, etc.) that need to be addressed through future measure development. We would urge interested readers to see the thoughtful critiques and guidance offered by Mestre et al. (2016) in their recent review on this subject (see Hughes, 2018; Irwing and Hughes, 2018 for guidance concerning measure development).

Regarding affect-related personality traits, there is an important need for further measure refinements. Our review of item level content combined with a number of empirical studies shows clear overlap between existing personality measures and personality trait EI measures. What we now need is a systematic study from the facet level that can identify which trait EI facets are unique and which are redundant duplications. In addition, it would be of interest to know which affect-related facets, if any, currently included within broad measures of personality (e.g., the NEO-PI-R) have been missed by specific measures (e.g., the TEIQue). These two lines of research would allow for the identification of much more comprehensive list of affect-related personality facets (see also Laborde and Allen, 2016; Petrides et al., 2016), which would be of great use to researchers and practitioners. The concept of EI competencies (e.g., Boyatzis, 2009) is redundant. Models and measures stemming from this perspective capture personality traits already accounted for, self-estimates of ability that are not relevant, and some broad emotion regulation strategies which are better considered within existing emotion regulation frameworks. Thus, we suggest that researchers interested in emotional skill or competence adopt measures of emotion regulation rather than problematic measures of social and emotional competence.

Fifth, the clear delineation of constructs raises the issue of nomenclature. We noted at the outset of this paper that logically speaking any construct labeled EI should consist of both emotion and intelligence. Currently, only the ability EI perspective meets this criterion and thus we would recommend that the label ‘EI’ is reserved exclusively for this perspective. We are far from the first authors to make such recommendations (e.g., Gignac, 2010), yet, despite previous calls, other EI-related characteristics have been resistant to change. Nevertheless, appropriate nomenclature is crucial for effective scientific communication and for reducing misconceptions. Thus we suggest that from now on, authors refer to ability EI (for affect-related intelligence), affect-related personality traits (for affect-laden personality measures such as those currently referred to as trait EI), and emotion regulation (for measures that concern goal-oriented use of emotions).

## Conclusion

In this review, we have outlined three theoretical perspectives on EI and proposed the theoretically driven Integrated Model of Affect-related Individual Differences as a stepping-stone toward building a greater understanding of EI-related individual differences and subsequent affective phenomena. This model posits that despite often being pitted against each other, the different perspectives are actually meaningfully entwined and coalesce to produce emotion-relevant behavior. We hope that our review and model will serve to guide future research and theoretical development. Although we should continue to be skeptical of EI, and actively criticize overblown claims based on atheoretical models and measures, we hope that this review demonstrates that all is not lost with regards to EI-related constructs.

## Author Contributions

Both authors contributed equally to the work and made a substantial, direct and intellectual contribution to the work. Both authors approved this manuscript for publication.

## Conflict of Interest Statement

The authors declare that the research was conducted in the absence of any commercial or financial relationships that could be construed as a potential conflict of interest.
